# The effect of neuraxial anesthesia on cancer recurrence and survival after cancer surgery: an updated meta-analysis

**DOI:** 10.18632/oncotarget.7683

**Published:** 2016-02-24

**Authors:** Meilin Weng, Wankun Chen, Wenting Hou, Lihong Li, Ming Ding, Changhong Miao

**Affiliations:** ^1^ Department of Anesthesiology, Fudan University Shanghai Cancer Centre, Department of Oncology, Shanghai Medical College Fudan University, Shanghai, China; ^2^ Department of Anesthesiology, Zhongshan Hospital, Fudan University, Shanghai, China

**Keywords:** neuraxial anesthesia, general anesthesia, cancer recurrence, survival, cancer outcome

## Abstract

Several animal and observational studies have evaluated the effects of neuraxial anesthesia on the recurrence and survival of cancer surgery; studies reported benefit, whereas others did not. To provide further evidence that neuraxial anesthesia(combined with or without general anesthesia (GA))may be associated with reduced cancer recurrence and long-term survival after cancer surgery, we conducted this meta-analysis. A total of 21 studies were identified and analyzed, based on searches conducted using PubMed, Web of Science, EMBASE database and the Cochrane Database of Systematic Reviews. After data abstraction, adjusted hazard ratios (HR) with 95% confidence intervals (CIs) were used to assess the impact of neuraxial anesthesia (combined with or without GA) and GA on oncological outcomes after cancer surgery. For overall survival (OS), a potential association between neuraxial anesthesia and improved OS (HR 0.853, CI 0.741-0.981, *P* = 0.026, the random-effects model) was observed compared with GA. Specifically, we found a positive association between neuraxial anesthesia and improved OS in colorectal cancer (HR 0.653, CI 0.430-0.991, *P* = 0.045, the random-effects model). For recurrence-free survival (RFS), a significant association between neuraxial anesthesia and improved RFS (HR 0.846, CI 0.718-0.998, *P* = 0.047, the random-effects model) was detected compared with GA. Our meta-analysis suggests that neuraxial anesthesia may be associated with improved OS in patients with cancer surgery, especially for those patients with colorectal cancer. It also supports a potential association between neuraxial anesthesia and a reduced risk of cancer recurrence. More prospective studies are needed to elucidate whether the association between neuraxial use and survival is causative.

## INTRODUCTION

Surgical resection is essential to the treatment of cancer. However, surgery itself has the potential to promote the development of metastases and reduce subsequent survival [1, 2]. Inhaled anesthetics and intravenous opioids may contribute to the suppression of cell-mediated immunity by decreasing the activity of natural killer (NK) cells [3-6]. Neuraxial anesthesia (including epidural anesthesia or spinal anesthesia) combined with or without general anesthesia (GA)can attenuate the neuroendocrine stress response and prevent immunosuppression. In addition, it also decreases the requirement for inhaled anesthetics and opioids [7, 8, 23, 24]. Therefore, neuraxial anesthesia may be beneficial for patients undergoing cancer surgery.

Recently, several retrospective studies for prostate and ovarian cancer have suggested a reduction in cancer recurrence and metastasis in patients receiving perioperative neuraxial analgesia [24, 28]. In contrast to these studies, the authors found that the use of neuraxial analgesia for perioperative pain control during colorectal cancer and prostate cancer surgery was not associated with a decreased cancer recurrence and metastasis [10, 12-14, 22, 25, 26]. Due to selection bias and other variation factors, the results of these findings are conflicting rather than conclusive.

Pei et al. have performed a meta-analysis to investigate the association between epidural anesthesia and prognosis of cancer patient after surgery [30]. However, they excluded several eligible studies (e.g., the studies by Scavonetto et al. [12], Lacassie et al. [16], Myles et al. [18], Binczak et al. [19], Lai et al. [20], Merquiol et al. [21], and Forget et al. [26]). To provide further evidence that neuraxial anesthesia (combined with or without GA)may be associated with reduced cancer recurrence and long-term survival after cancer surgery, we conducted this meta-analysis.

## RESULTS

### Basic characteristics

After a careful and thorough search, 21 eligible studies [9-29] met the inclusion criteria (Table 1). These studies included approximately 15,160 cases in the neuraxial anesthesia group and approximately 36,460 cases in the GA group. The studies were published between 2004 and 2014. Overall survival (OS) and recurrence-free survival (RFS) were defined as two end points, respectively. OS was defined from surgery to death for any reason. RFS was defined from surgery to the first occurrence of disease progression or relapse due to the primary cancer.

**Table 1:** Characteristics of eligible studies for meta-analysis

Author	Year	Cancer type	Design type	Survival	neuraxial	No neuraxial	HR	95%CI	Quality*
R.Christopherson-I [9]	2008	Non- metastaticColon cancer	prospective	Overall survival	85	92	0.216	0.065-0.718	7
R.Christopherson-II [9]	2008	MetastaticColon cancer	prospective	Overall survival	85	92	0.699	0.395-1.236	7
K.C. Cummings [10]	2011	Colorectal cancer	retrospective	Overall Survival	9670	32481	0.91	0.87-0.94	8
A.Gupta-I [11]	2011	Colon caner	retrospective	Overall Survival	562	93	0.82	0.3-2.19	6
A.Gupta-II [11]	2011	rectal caner	retrospective	Overall Survival	562	93	0.45	0.22-0.90	6
F.Scavonetto [12]	2004	Prostate cancer	retrospective	All cause death	1642	1642	1.32	1.00-1.74	8
P.Y.Wuethrich [13]	2010	Prostate cancer	retrospective	Overall survival	103	158	0.61	0.29-1.28	7
P.Y.Wuethrich [14]	2013	Prostate cancer	retrospective	Overall survival	67	81	1.79	0.95-3.39	7
L.Lin [15]	2011	Ovarian cancer	retrospective	Overall survival	106	37	0.824	0.699-0.930	6
H.J.Lacassie [16]	2013	Ovarian cancer	prospective	Overall survival	37	43	0.73	0.36-1.52	6
J.G.Hiller [17]	2014	Gastro-oesophageal cancer	retrospective	Overall survival	97	43	0.42	0.21-0.83	7
P.S.Myles [18]	2011	Abdominal cancer	prospective	Overall survival	230	216	0.95	0.77-1.18	7
M. Binczak [19]	2013	Abdominal cancer	retrospectivve	Overall survival	69	63	0.69	0.43-1.09	6
R.Lai [20]	2011	Hepatocellular caner	retrospective	Overall survival	62	117	1.26	0.81-1.97	7
F.Merquiol [21]	2013	Laryngeal and hypopharyngeal cancer	retrospective	Overall survival	111	160	0.61	0.39-0.96	7
A.Gottschalk [22]	2010	Colorectal cancer	retrospective	Recurrence free survival	256	253	0.82	0.49-1.35	7
K.C.Cummings [10]	2011	Colorectal cancer	retrospective	Recurrence free survival	9670	32481	1.05	0.95-1.15	8
A.K.Exadaktylos [23]	2006	Breast cancer	retrospective	Recurrence free survival	50	79	0.21	0.06-0.71	7
F.Scavonetto [12]	2004	Prostate cancer	retrospective	Recurrence free survival	1642	1642	1.00	0.83-1.21	8
B.Biki [24]	2008	Prostate cancer	retrospective	Recurrence free survival	102	123	0.43	0.22-0.83	6
B.C.H.Tsui [25]	2010	Prostate cancer	prospective	Disease free survival	49	50	1.33	0.64-2.77	6
P.Forget [26]	2010	Prostate cancer	retrospective	Recurrence free survival	578	533	0.84	0.52-1.17	7
P.Y.Wuethrich [13]	2010	Prostate cancer	retrospective	Recurrence free survival	103	158	1.14	0.84-1.54	7
P.Y.Wuethrich [14]	2013	Prostate cancer	retrospective	Distant Recurrence free survival	67	81	0.58	0.27-1.29	7
K.S.Tseng [27]	2014	Prostate cancer	retrospective	Recurrence free survival	1166	798	0.91	0.70-1.18	7
G.S.de.Oliveira.Jr-I [28]	2011	Ovarian cancer	retrospective	Recurrence free survival	26	127	0.37	0.19-0.73	7
G.S.de.Oliveira.Jr-II [28]	2011	Ovarian cancer	retrospective	Recurrence free survival	29	127	0.86	0.52-1.41	7
H.J.Lacassie [16]	2013	Ovarian cancer	prospective	Recurrence free survival	37	43	0.73	0.40-1.31	6
P.S.Myles [18]	2011	abdominal	prospective	Recurrence free survival	230	216	0.95	0.76-1.17	7
M.Binczak [19]	2013	Abdominal cancer	retrospective	Recurrence free survival	69	63	0.81	0.52-1.27	6
J.G.Hiller [17]	2014	Gastro-oesophageal cancer	retrospective	Recurrence free survival	97	43	0.33	0.17-0.63	7
R.Lai [20]	2011	Hepatocellular cancer	retrospective	Recurrence free survival	62	117	4.31	2.24-8.29	7
F.Merquiol [21]	2013	Laryngeal and hypopharyngeal cancer	retrospective	Cancer free survival	111	160	0.49	0.25-0.96	7
H.Ismail [29]	2010	Cervical cancer	retrospective	Local or systemic recurrence	63	69	0.95	0.54-1.67	6

* evaluated by the 9-star Newcastle-Ottawa Scale.

There were 15 studies [9-21] that involved OS, 7 of which [9-12, 15, 17, 21] demonstrated a positive relationship between neuraxial anesthesia and improved OS. Five studies [9-11] were on colorectal cancer, and 3 studies [12-14] were on prostate cancer. The remaining cancer types investigated were ovarian cancer, gastro-oesophageal cancer, laryngeal and hypopharyngeal cancer, abdominal cancer, and hepatocellular cancer. [15-21]

There were 19 studies [10, 12-14, 16-29] that involved RFS, 6 of which [17, 20, 21, 23, 24, 28] showed a positive relationship between neuraxial anesthesia and improved RFS. Seven studies [12-14, 24-27] were on prostate cancer, and 3 studies [16, 28] were on ovarian cancer. The remaining cancer types investigated were breast cancer, colorectal cancer, gastro-oesophageal cancer, abdominal cancer, laryngeal and hypopharyngeal cancer, hepatocellular cancer, and cervical cancer [17-21, 29].

### Association between neuraxial anesthesia and OS

Significant heterogeneity was detected in the HRs for OS (heterogeneity chi-squared = 36.16, *P* = 0.001, I-squared = 61.3%), so the random-effects model was used to analyze these data. A significant association between neuraxial anesthesia and improved OS was then observed compared with GA (HR 0.853, CI 0.741-0.981, *P* = 0.026) (Table 2, Figure 2A). The further sensitivity analysis also proved that our results were robust and stable (Figure 3A). Because 5 studies [9-11] were on colorectal cancer, a subgroup analysis was conducted specifically for them. An obvious association between neuraxial anesthesia and improved OS was found in colorectal cancer (HR 0.653, CI 0.430-0.991, *P* = 0.045) (Table 2, Figure 2B). No significant association between neuraxial anesthesia and improved OS was detected in prostate cancer [12-14] (Table 2).

**Figure 1:**
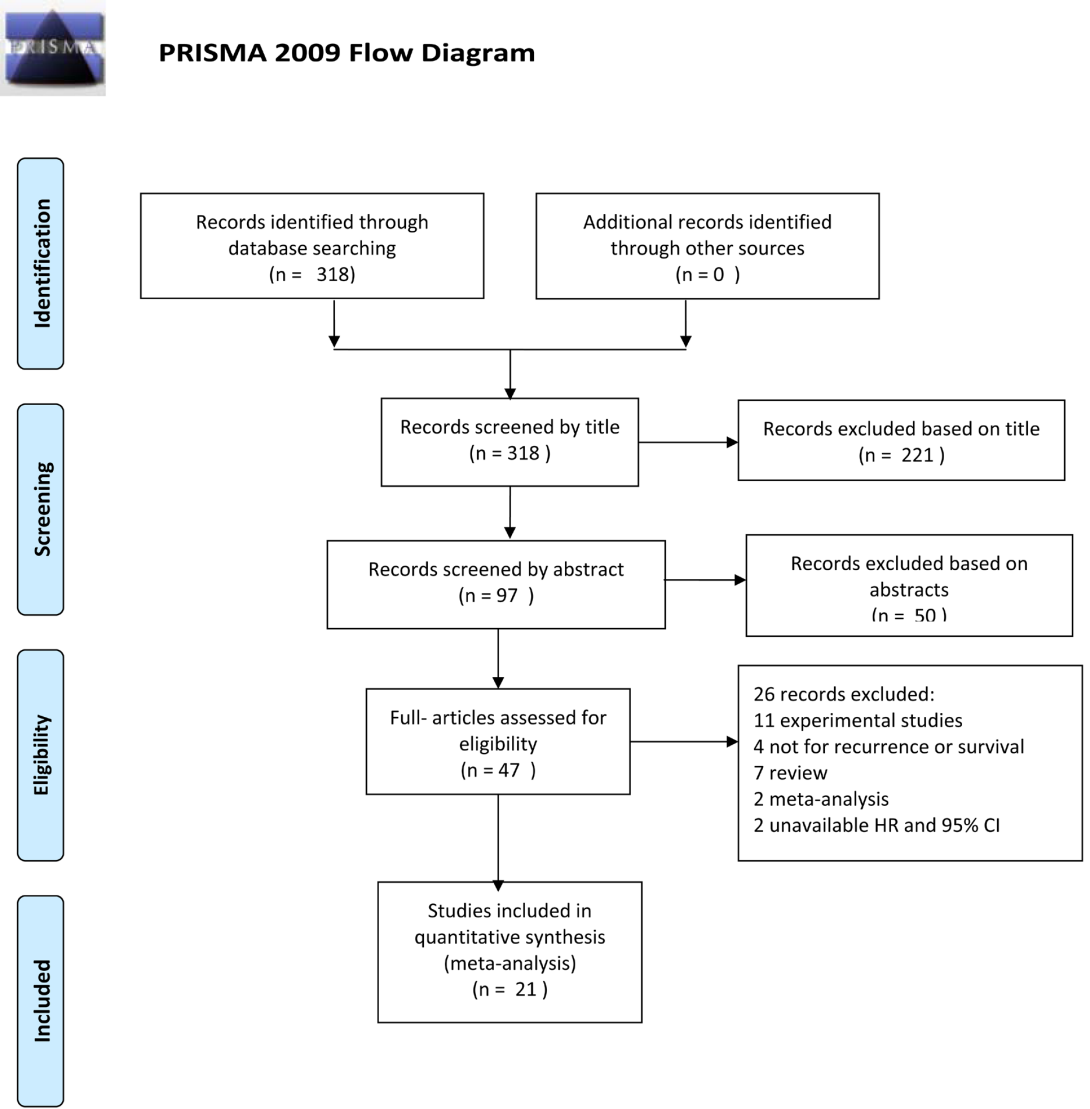
PRISMA 2009 flow diagram

**Figure 2:**
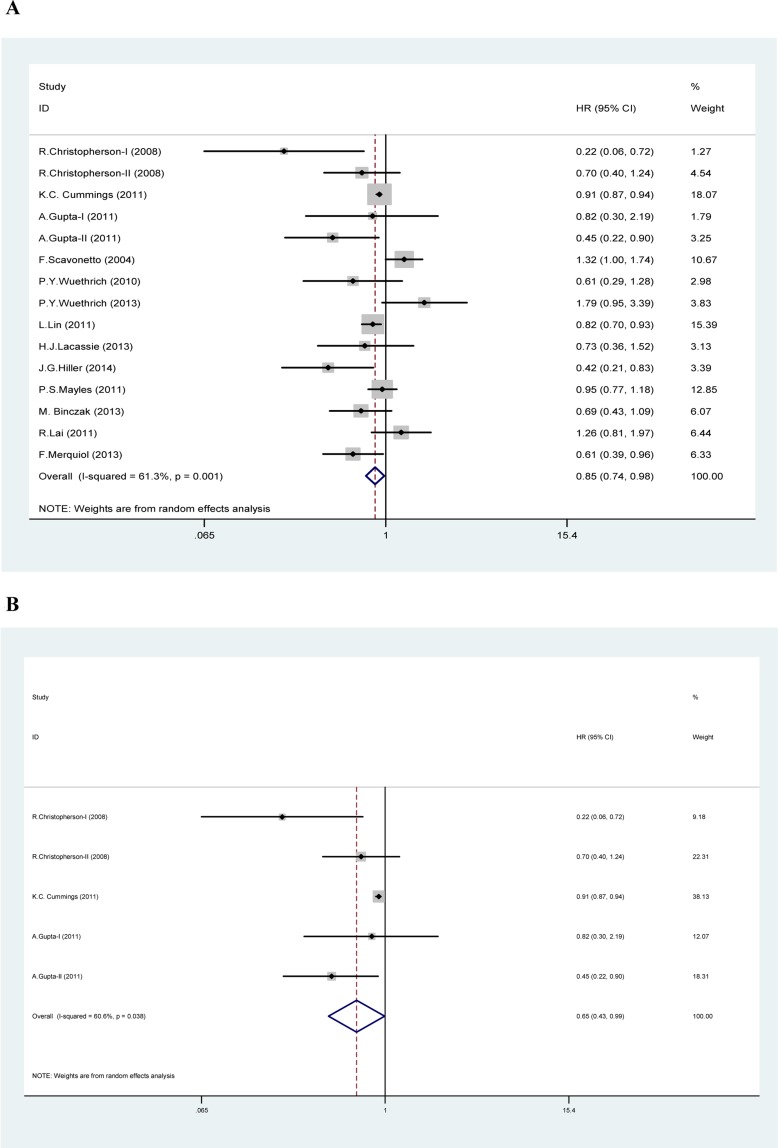

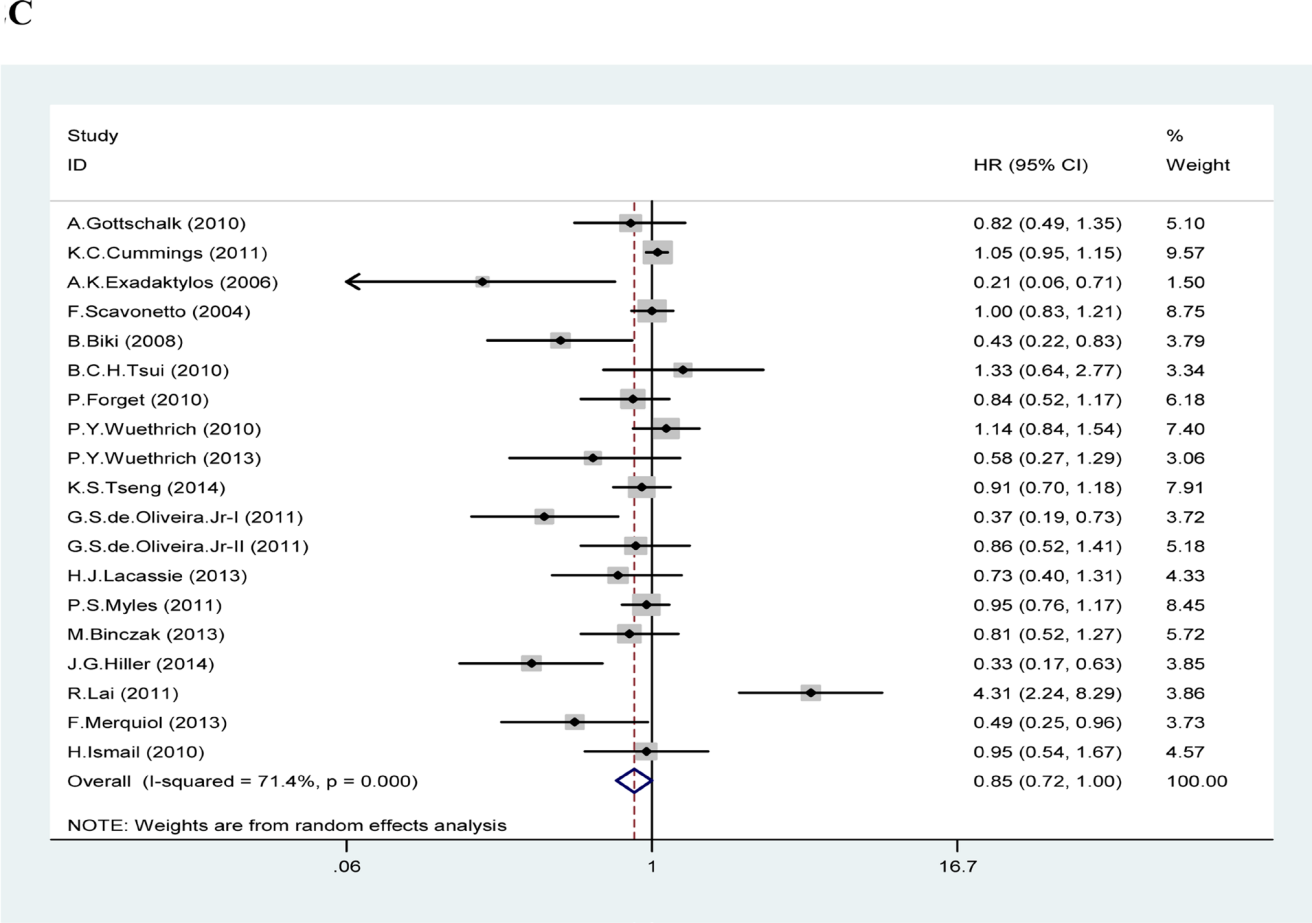
Forest plot of meta-analysis In Figure 2A (overall survival analysis), 2B (overall survival analysis in colorectal cancer) and 2C (recurrence-free survival analysis), each study is shown by the point estimate of the hazard ratio (HR) and 95% confidence interval (CIs).

**Figure 3:**
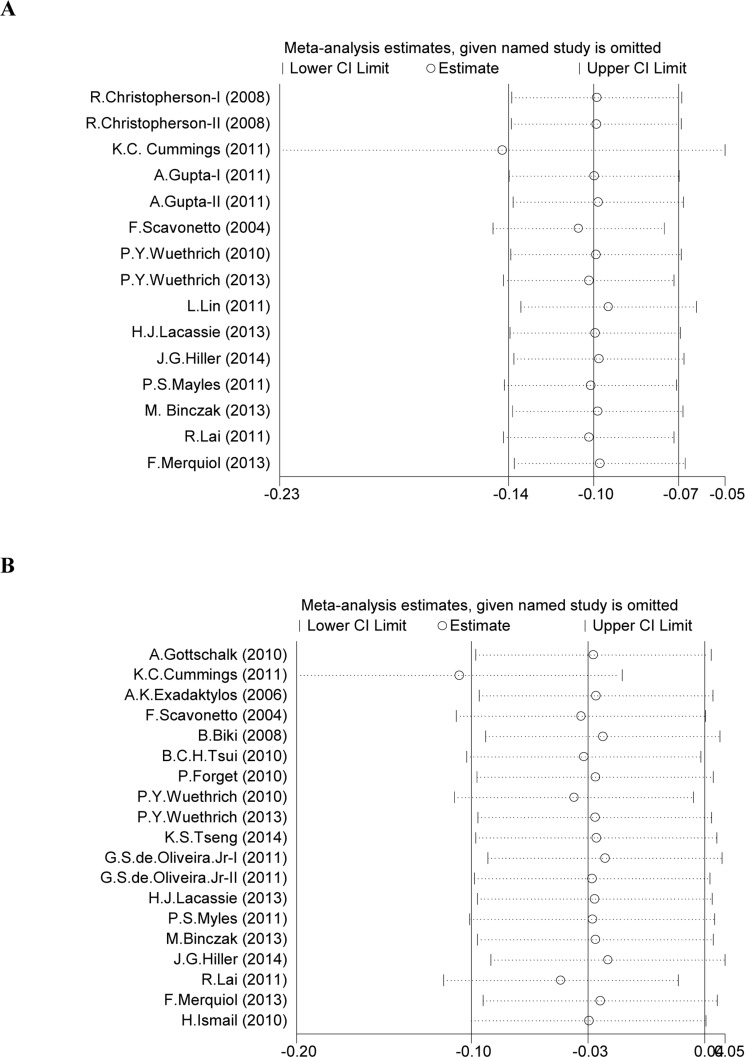
Sensitivity analysis of meta-analysis Figure 3A shows the influence of individual studies on the summary HR for OS. Figure 3B shows the influence of individual studies on the summary HR for RFS.

**Table 2:** Pooled hazard ratios for overall survival and recurrence-free survival

Pooled analysis	Study number	HR (95% CI)	P for difference	P for heterogeneity and I-squared
**Overall Survival**				
All groups	15	0.853 (0.741-0.981)	0.026	0.001 and 61.3%
In colorectal cancer	5	0.653 (0.430-0.991)	0.045	0.038 and 60.6%
In prostate cancer	3	1.194 (0.735-1.941)	0.474	0.085 and 59.5%
**Recurrence-free Survival**				
All groups	19	0.846 (0.718-0.998)	0.047	0.000 and 71.4%
In prostate cancer	7	0.919 (0.765-1.104)	0.366	0.127 and 39.7%
In ovarian cancer	3	0.640 (0.396-1.033)	0.068	0.133 and 50.4%

### Association between neuraxial anesthesia and RFS

Apparent heterogeneity was observed in the HRs for RFS (heterogeneity chi-squared = 62.98, *P* = 0.000, I-squared = 71.4%), so the random-effects model was used to analyze these data. A potential association between neuraxial anesthesia and improved RFS was then observed compared with GA (HR 0.846, CI 0.718-0.998, *P* = 0.047) (Table 2, Figure 2C). A further sensitivity analysis also indicated that our results were robust and stable (Figure 3B). Subgroup analyses were performed by prostate cancer [12-14, 24-27] and ovarian cancer [16, 28]. No significant association between neuraxial anesthesia and improved RFS was found in prostate cancer and ovarian cancer (Table 2).

### Publication bias

**Figure 4:**
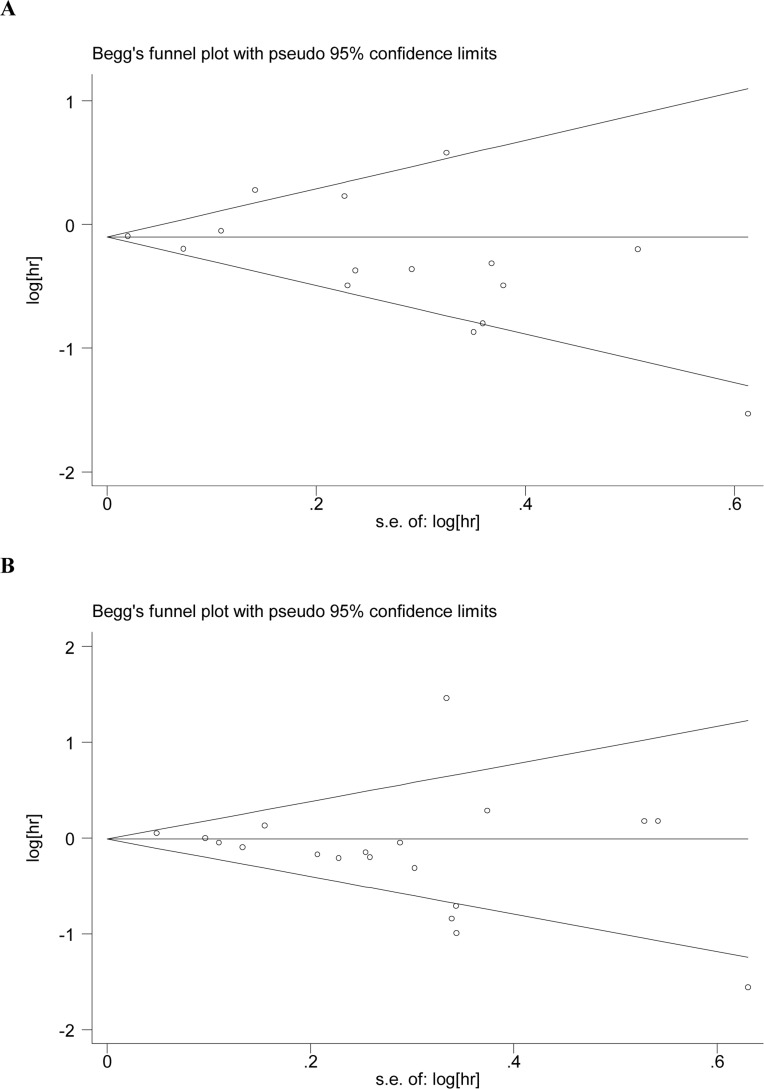
Publication bias plots Figure 4A and 4B show the Begg's test funnel plots of studies included in the meta-analysis for OS and RFS.

The funnel plot was used to evaluate publication bias. Moreover, Egger's test and Begg's test showed no evidence of publication bias in OS (*P* = 0.198 and *P* = 0.181, respectively), and RFS (*P* = 0.142 and *P* = 0.132, respectively) (Figure 4A, 4B).

## DISCUSSION

Our results suggest that neuraxial anesthesia maybe associated with improved OS in patients with cancer surgery, especially for those patients with colorectal cancer. The impact of neuraxial anesthesia on recurrence and survival after cancer surgery has been a contentious issue [9-21]. The use of neuraxial anesthetic techniques has been found to be associated with improved OS after colorectal cancer, prostate cancer, gastro-oesophageal cancer, laryngeal and hypopharyngeal cancer, and ovarian cancer surgery [9-12, 15, 17, 21]. Some recently published studies, however, did not find any difference in mortality after colorectal cancer, prostate cancer, ovarian cancer, abdominal cancer, and hepatocellular cancer surgery in patients with neuraxial anesthesia compared with those without [9, 11, 13, 14, 16, 18-20]. Our results are consistent with those in a previous meta-analysis in our institution by Chen et al., which confirmed the positive effect of neuraxial anesthesia on cancer survival [37].

Host defense is established as the primary determinant of progression of cancer, [7] and the function of NK cells is the most important component for recognizing and killing tumor cells [38]. Multiple surgical factors, such as surgical trauma, inflammation, pain, anesthetics, blood transfusion, ischemia, etc., can negatively affect the balance between metastasis and immune surveillance in the perioperative period [39, 40, 41]. However, each effect can be modulated by neuraxial anesthesia, which can help to preserve immune function [1, 2]. The activation of the autonomic nervous system and the hypothalamic-pituitary-adrenal axis (HPA axis) releases more neuroendocrine stress, which may lead to immunosuppression during the perioperative period [42, 43]. Surgery also can reduce NK cell numbers, suppress immunity, facilitate the growth of preexisting micro-metastases, and disseminate malignant cells during tumor manipulation [44, 45]. Anesthesia may impair numerous functions, including neutrophil, macrophage, dendritic cell, T lymphocyte, and NK cell functions [46]. Opioids can produce immunosuppression by reducing NK cells, inhibiting lymphocyte proliferation, and altering cytokine expression [4, 5]. They can also promote angiogenesis-dependent tumor growth through the μ receptors present on endothelial cells [47]. Neuraxial anesthesia can attenuate neuroendocrine stress by cutting off afferent neural transmission from reaching the central nervous system and blocking descending efferent activation of the sympathetic nervous system [42, 43, 48]. It can reduce anesthetic requirements, decrease the release of endogenous opioids, improve tissue oxygenation, and promote innate anti-tumor factors through the effects of local anesthetic [7, 8, 49, 50]. It can also bring early survival benefit by reducing the incidence of thrombotic events, infection, cardiovascular and pulmonary complications [51, 52]. Therefore, neuraxial anesthesia (combined with or without GA) may bring about better outcomes regarding tumor growth and metastasis.

Our results suggest that neuraxial anesthesia maybe associated with improved OS in patients with cancer surgery, especially for those patients with colorectal cancer. However, studies in patients undergoing colorectal surgery have found inconsistent results. Christopherson et al. [9-11, 53] found that epidural anesthesia may improve survival among patients with non-metastatic colon cancer before 1.46 years, but it had no impact on patient survival with metastatic colon cancer [9]. Gupta et al. found a reduction in all-mortality after rectal cancer but not colon cancer in patients with epidural usage [11]. Cummings et al. showed that epidural use is associated with improved survival in patients with colorectal cancer, but an association between epidural use and decreased cancer recurrence is not supported [10]. However, another study did not identify any benefit with epidural analgesia for OS after laparoscopic colorectal resection [53]. Our results are also in accordance with those from a previous meta-analysis in our institution by Chen et al., which confirmed the beneficial impact of neuraxial anesthesia on colorectal cancer survival [37]. We speculate that colorectal cancer may be more sensitive to the immune protective effect of neuraxial anesthesia.

Our results also suggest a significant association between neuraxial anesthesia and reduced cancer recurrence. Neuraxial anesthesia has been reported to decrease the recurrence rate after surgery for breast cancer (paravertebral block), prostate cancer (thoracic epidural analgesia), ovarian cancer (epidural anesthesia/analgesia), hepatocellular cancer (epidural anesthesia), laryngeal and hypopharyngeal cancer (cervical epidural anesthesia and analgesia), and gastro-oesophageal cancer (epidural analgesia) [17, 20, 21, 23, 24, 28], whereas other studies reported no association between neuraxial anesthesia and cancer recurrence [10, 12-14, 16, 18, 19, 22, 25-29]. Our results are in contrast to those in two previous meta-analyses by Chen et al. and Pei et al [30, 37]. We could not confirm the results observed by Pei et al., as they failed to include all eligible studies available at the time of analysis. Their results' lack of significance is likely due to the limited study number. In the two years since that analysis, a great number of clinical studies focusing on the association between neuraxial anesthesia and oncological outcome have emerged. For our current study, more prospective and retrospective studies were collected to assess this important clinical problem. Our meta-analysis is an update to the study by Chen et al.

Our study does have some unavoidable limitations. First, different types of cancer have different tumor biological characteristics (e.g., the type and the site of tumor, cancer staging and adjuvant therapy), and whether our conclusions can be applied to them all is unknown. Second, the limited number of studies makes the results of subgroup analysis less reliable. Moreover, there is only one study for some cancers, such as breast cancer, gastro-esophageal cancer, laryngeal and hypopharyngeal cancer, cervical cancer, and hepatocellular carcinoma. Third, some other confounding variables are not controlled in this meta-analysis, such as different surgical techniques, difficulty in defining relapse, various patient populations, different length of follow-up and various timing of epidural use. Fourth, our systemic review is also restricted by the nonrandomized and retrospective nature of the studies. Moreover, we only selected studies published in English, which would lead to so-called “English language bias” that may reduce the accuracy of our results.

In conclusion, our meta-analysis suggests that neuraxial anesthesia may be associated with improved OS in patients with cancer surgery, especially for these patients with colorectal cancer. Our results also support a potential association between neuraxial anesthesia and a reduced risk of cancer recurrence. Our finding should be interpreted with considerable caution, and more prospective studies are needed to elucidate whether neuraxial anesthesia has an effect on cancer-specific outcome in patients undergoing cancer surgery.

## MATERIALS AND METHODS

### Study identification and data extraction

Literature was retrieved through the PubMed, Web of Science, EMBASE database, and the Cochrane Database of Systematic Reviews (updated to August-1, 2015) using the following keywords: (i) “neuraxial anesthesia,” “epidural anesthesia,” “spinal anesthesia,” “regional anesthesia,”“anesthetic technique,” or “general anesthesia,” and (ii) “recurrence,” “metastasis,” “survival,” or “prognosis,” and (iii) “neoplasm,”“cancer,” or“carcinoma”. Only studies published in English were included. Both abstracts and full text papers were eligible. We did not define the minimum number of patients to be included for this meta-analysis. Three hundred and eighteen papers were screened out by this strategy. After reviewing their titles, we identified 97 papers for further consideration. And after reading their abstracts, we finally reviewed 47 potentially eligible papers by full text reading. The study flowchart is shown in Figure 1.

The included studies should meet the following criteria: (i) evaluating the effect of neuraxial anesthesia (combined with or without GA) and GA on oncological outcome after cancer surgery, (ii) independent prospective or retrospective study, and (iii) having hazard ratios (HR) with 95% confidence intervals (CIs) (or having adequate available data to calculate). As most other studies suggested [31, 32], we extracted the HR adjusted for other potentially suspected factors. The adjusted HRs, rather than crude odd ratios or relative risks, might be more dependable to reflect the impact of anesthetic technique on oncological outcome. As a result, we identified 21 eligible studies for this systemic review. The following variables were extracted from each study if available: first author's name, publication year, cancer type, design type, survival type, numbers in neuraxial anesthesia group, numbers in GA group, and HR with 95% CIs of treatment outcomes.

Two authors (M.L.W and W.K.C) collected the information carefully and independently. We used the 9-star Newcastle-Ottawa Scale to evaluate the study quality (The Newcastle-Ottawa Scale for assessing the quality of nonrandomized studies in systemic review. [33] Ottawa, Canada: Dept. of Epidemiology and Community Medicine, University of Ottawa. http://www.ohri.ca/programs/clinical_epidemiology/oxford.htm. Accessed on 2015 Jan 1).

### Statistical analysis

This systematic review and meta-analysis was carried out in compliance with the PRISMA statement for reporting meta-analysis [34, 35]. For each study, HR along with its 95% CIs was recorded to evaluate the association between neuraxial anesthesia and oncological outcomes. The heterogeneity among studies was checked by Cochran chi-square Q statistics or I^2^ statistics, which decided the use of fixed-effects model or random-effects model. When *P*-value < 0.05 or I-square > 25%, heterogeneity was considered and the random-effects model was chosen to calculate HR using the DerSimonian and Laird method. Otherwise, the fixed-effects model was applied using the Mantel–Haenszel method [36]. We conducted subgroup analysis according to cancer type. We also performed sensitivity analysis by omitting each study to find potential outliers. The publication bias was examined visually in a funnel plot of In [OR] against its standard error (SE), and the degree of asymmetry was tested using Egger's test and Begg's test. A symmetrical plot (*P*-value > 0.05)suggested no publication bias. All of the statistical analyses were calculated using Stata/SE version 12.0 (Stata Corporation, College Station, TX).
